# Optical Properties and Applications of Diffraction Grating Using Localized Surface Plasmon Resonance with Metal Nano-Hemispheres

**DOI:** 10.3390/nano14191605

**Published:** 2024-10-05

**Authors:** Tomoya Kubota, Shogo Tokimori, Kai Funato, Hiroaki Kawata, Tetsuya Matsuyama, Kenji Wada, Koichi Okamoto

**Affiliations:** 1 Department of Physics and Electronics, Osaka Metropolitan University, Sakai-shi 599-8531, Osaka, Japankawata_hiroaki@omu.ac.jp (H.K.); matsuyama.tetsuya@omu.ac.jp (T.M.); 2 Equipment Sharing Center for Advanced Research and Innovation, Osaka Metropolitan University, Sakai-shi 599-8531, Osaka, Japan; wada.kenji@omu.ac.jp

**Keywords:** plasmonics, localized surface plasmon resonance, metal nano-hemisphere grating, diffraction grating, third-order nonlinear laser spectroscopy

## Abstract

This study investigates the optical properties of diffraction gratings using localized surface plasmon resonance (LSPR) with metal nano-hemispheres. We fabricated metal nano-hemisphere gratings (MNHGS) with Ga, Ag, and Au and examined their wavelength-selective diffraction properties. Our findings show that these gratings exhibit peak diffraction efficiencies at 300 nm, 500 nm, and 570 nm, respectively, corresponding to the LSPR wavelengths of each metal. The MNHGs were created through thermal nanoimprint and metal deposition, followed by annealing. The experimental and simulation results confirmed that the MNHGs selectively diffract light at their resonance wavelengths. Applying these findings to third-order nonlinear laser spectroscopy (MPT-TG method) enhances measurement sensitivity by reducing background noise through the selective diffraction of pump light while transmitting probe light. This innovation promises a highly sensitive method for observing subtle optical phenomena, enhancing the capabilities of nonlinear laser spectroscopy.

## 1. Introduction

Localized surface plasmon resonance (LSPR) is a phenomenon that occurs in nano-sized metal structures, which are on the order of, or smaller than, the wavelength on which the energy of light is confined, leading to the excitation of plasmon resonance [1]. This phenomenon results in the generation of a strong localized electric field on the surface of the metal nanostructure, significantly enhancing the emission, absorption, and scattering of nearby materials [1,2]. Additionally, it is known that the resonance conditions of LSPR depend on the type, size, and shape of the metal nanostructure [2]. A significant characteristic of the metal nanostructure is that it does not interact with light outside the resonance wavelength.

In this study, we proposed and defined a novel structure called a metal nano-hemisphere grating (MNHG), in which metal nano-hemispheres are arranged in a grating pattern. This paper investigates the optical properties of this MNHG through both simulation and experimentation. We demonstrated wavelength selectivity, showing that the grating acts within a limited wavelength range due to the resonance characteristics of the metal nano-hemisphere structure.

We explore how the diffraction grating enhances and improves third-order nonlinear laser spectroscopy. Third-order nonlinear laser spectroscopy is a versatile method that enables the observation of various phenomena, such as thermal dynamics [3,4] and electron transfer [5,6]. This technique has been applied in a wide range of studies, including the analysis of ultrafast thermal conductance [3], time-resolved fluorescence [6], and single-pulse nonlinear Raman spectroscopy [5]. Additionally, it is integral to the field of single-cycle nonlinear optics [7] and is underpinned by the principles outlined in foundational texts on nonlinear optics [8]. Various approaches, such as laser-induced dynamic grating [9], have also been developed to probe transient processes using this technique. 

**Figure 1:**
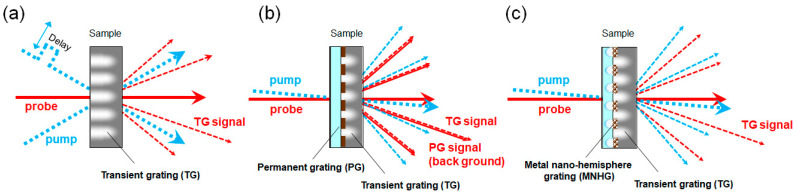
Schematic diagrams illustrating the alignment and signal generation in different transient grating (TG) techniques. (**a**) The traditional transient grating (TG) technique, where pump beams create an interference pattern, and the TG signal is detected through the diffraction of a probe beam. (**b**) The mask pattern transferred transient grating (MPT-TG) technique, where a metal thin-film grating generates the interference pattern, leading to TG signal detection alongside significant background signals (PG signal). (**c**) The proposed metal nano-hemisphere grating (MNHG) technique, utilizing LSPR to achieve wavelength-selective diffraction. This setup reduces background signals, allowing for more precise TG signal detection, thereby enhancing measurement sensitivity.

Figure 1a shows a spectroscopic method known as the transient grating (TG) technique. This method generates an interference pattern with two laser beams (pump beams), inducing third-harmonic nonlinear polarization in the sample [10,11,12,13,14]. The detection of this third-harmonic nonlinear polarization is achieved through the diffraction of another laser beam (probe beam), allowing for the investigation of changes in optical properties [15,16,17,18]. This technique requires the maintenance of coherence among three laser beams; therefore, a complex optical system and sophisticated adjustments are needed for generating the interference pattern.

Figure 1b shows the third-order nonlinear laser spectroscopy method called the mask pattern transferred transient grating (MPT-TG) technique [19,20]. This technique utilizes a metal thin-film grating to generate an interference pattern, eliminating the need for a complex optical system and advanced adjustments. The technique offers simple setup and easy alignment. However, a limitation of the metal thin-film grating is its lack of wavelength selectivity. As a result, the probe beam not only diffracts due to the third-harmonic nonlinear polarization but also due to the diffraction grating, leading to the detection of significant background signals [19,20,21]. 

In this study, we introduce wavelength selectivity to the diffraction grating with metal thin-film by leveraging the LSPR of the metal nano-hemispheres. The configuration of our spectroscopy method is shown in Figure 1c. In the MPT-TG method using the MNHG, since the diffraction grating has wavelength characteristics, it is anticipated that measurements with reduced background and enhanced sensitivity can be achieved, unlike the MPT-TG method using a metal thin-film grating. This paper reports the fabrication methods of a MNHG with metal nano-hemispheres arranged in a diffraction grating pattern and the results of the investigation into its optical properties through simulation and experimentation.

Third-order nonlinear spectroscopy is a powerful tool for detecting subtle changes in refractive index and extinction coefficient induced by light irradiation, making it applicable for observing a wide range of phenomena, including electron dynamics, molecular dynamics, thermal dynamics, and chemical reactions. The method we propose, which incorporates MNHGs, enables stable and accurate measurements with a simple and easy-to-implement setup, eliminating the need for complex beam alignment and phase control. The use of MNHGs significantly reduces background noise, thereby enhancing the signal-to-noise ratio and improving detection sensitivity. Moreover, the ability to tailor the diffraction grating’s response to specific wavelengths introduces versatility in studying various materials and phenomena in nonlinear laser spectroscopy. 

## 2. Materials and Methods

**Figure 2:**
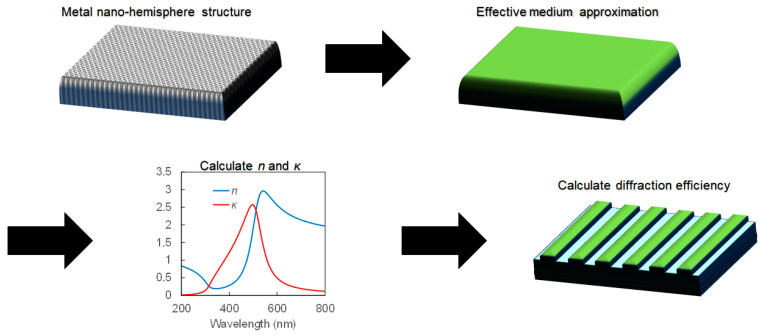
Overview of the simulation process for determining the diffraction efficiency of the metal nano-hemisphere grating (MNHG). The figure illustrates the step-by-step procedure, starting with the measurement of transmission through a sample with metal nano-hemispheres, followed by the calculation of the extinction spectrum, and concluding with the determination of the diffraction efficiency using the effective medium approximation (EMA) and finite-difference time-domain (FDTD) simulations. The key parameters involved in the simulations are also shown.

Figure 2 shows the process of determining the diffraction efficiency of the MNHG through simulation. The transmission of a sample featuring metal nano-hemispheres was first measured across its entire surface followed by the calculation of the extinction spectrum using the following equation:

(1) $$A=-\log_{10}\left(\frac{I}{I_{0}}\right)$$

To obtain a nearly identical spectrum to the acquired extinction spectrum, five parameters (*ε*_∞_, *ε*_b_, *γ*, *ω*_p_^2^) were set using the effective medium approximation (EMA) method with finite-difference time-domain (FDTD) simulation. FDTD simulations were performed using commercially available software (Poynting for Optics, Fujitsu, Kanagawa, Japan). Periodic and absorption boundary conditions were applied in the X and Y directions and in the Z direction, respectively. Pulsed light, consisting of a differential Gaussian function with a pulse width of 0.5 fs and an electric field of 1 V/m, was irradiated under an X-polarized source. The peak position of the excitation pulse spectrum was approximately 600 THz (wavelength: 500 nm). Using the five parameters (*ε*_∞_, *ε*_b_, *γ*, *ω*^2^, *ω*_p_^2^) and Equations (2) and (3), the real part *ε*_1_ and the imaginary part *ε*_2_ of the dielectric function were determined. Subsequently, the effective refractive index *n* and extinction coefficient *κ* of the metal nano-hemisphere structure were calculated using Equations (4) and (5) based on the obtained dielectric function.

(2) $$\varepsilon_{1}=\varepsilon_{b}+\frac{\omega_{p}^{2}\left(\omega^{2}-\omega_{0}^{2}\right)}{\left[{\left(\omega^{2}-\omega_{0}^{2}\right)}^{2}+\gamma^{2}\right]}$$

(3) $$\varepsilon_{2}=\frac{\omega_{p}^{2}\omega_{0}\gamma }{\left[{\left(\omega^{2}-\omega_{0}^{2}\right)}^{2}+\gamma^{2}\right]}$$

(4) $$n=\frac{\sqrt{\varepsilon_{1}+\sqrt{{\varepsilon_{1}}^{2}+{\varepsilon_{2}}^{2}}}}{\sqrt{2}}$$

(5) $$\kappa =\frac{\sqrt{-\varepsilon_{1}+\sqrt{{\varepsilon_{1}}^{2}+{\varepsilon_{2}}^{2}}}}{\sqrt{2}}$$

where *ε*_b_ represents the background permittivity, *ω*_p_ is the plasmon frequency, and *γ* is the damping coefficient. Additionally, the diffraction efficiency of the diffraction grating *η* can be expressed using the absorption coefficient α and the thickness of the diffraction grating *d* in Equation (6) [6,19];

(6) $$\eta ={\left(\frac{\pi d\Delta n}{\lambda }\right)}^{2}+{\left(\frac{\Delta \alpha d}{4}\right)}^{2}$$

The absorption coefficient α can be expressed using extinction coefficient *κ* in Equation (7), so the diffraction efficiency *η* can be expressed in Equation (8) based on Equations (4)–(7).

(7) $$\alpha =\frac{4\pi \kappa }{\lambda }$$

(8) $$\eta ={\left(\frac{\pi d}{\lambda }\right)}^{2}\left(\Delta n^{2}+4\Delta \kappa^{2}\right)$$

This equation shows that *η* is proportional to Δ*n*^2^ + 4Δ*κ*^2^, which is derived solely from the optical constants. Δ represents the difference in refractive index and extinction coefficient between the metal nano-hemisphere and the adjacent material. In this study, simulations were conducted using three types of metals: Ga, Ag, and Au, to investigate the optical properties resulting from the use of these metals.

**Figure 3:**
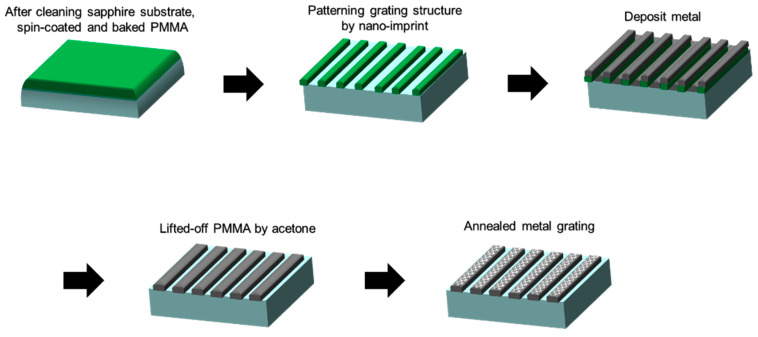
Step-by-step fabrication process of the metal nano-hemisphere grating (MNHG) structure. The figure outlines the key stages, starting with the spin-coating of PMMA onto a sapphire substrate, followed by thermal nanoimprint to form the diffraction grating pattern. After metal deposition and lift-off, the final MNHG structure is obtained through precise heating under a nitrogen atmosphere. Each step is illustrated to provide a clear overview of the method used to create the MNHG structure.

Figure 3 shows the process of fabricating the MNHG structure. First, PMMA dissolved in cyclohexanone, (45 g cyclohexanone, 3.5 g PMMA, 67 cP) was spin-coated onto a cleaned sapphire substrate (slope: 5 s, 4000 rpm: 90 s, slope: 5 s). Second, the resist was heated at 180 °C for 60 min using an electric furnace (Yamato Scientific, FO100, Tokyo, Japan) to evaporate the cyclohexanone and induce a glass transition in PMMA. Third, the glass-transitioned PMMA was shaped into a diffraction grating using a thermal nanoimprint device (Litho Tech Japan Corporation, LTNIP-550, Saitama, Japan). The mold used had a pitch of 4 μm, a structural depth of 1 μm, and a structure area of 5.0 × 5.0 mm, with a mold area of 10 × 10 mm in a line and space configuration. The imprint conditions involved heating the mold to 170 °C, applying an imprint force of 0.8 kN for 15 min, and then cooling with water until it reached approximately 40 °C while maintaining pressure. Fourth, the cross-section of the imprinted sample was observed using a scanning electron microscope (SEM) (FlexSEM 1000 II, Hitachi High-Tech, Tokyo, Japan) to confirm the thickness of the residual film. Fifth, the remaining resist was then removed using a reactive ion etching apparatus (SUMCO Corporation, RIE-10NR-OFU, Tokyo, Japan). In this experiment, oxygen was introduced at a rate of 50 sccm, and the process was carried out for only 1 min. By carrying out these steps, a grating pattern of PMMA resist was fabricated.

After fabricating the PMMA resist, thin films of Ga, Ag, and Au were deposited onto the PMMA resist using resistive heating evaporation (SVC-700TM, Sanyu Electron, Tokyo, Japan). Once the deposition was complete, the samples were immersed in a beaker filled with acetone, covered with aluminum foil, and left undisturbed for about 20 min. After that, ultrasonic cleaning was performed for 5 s to remove the PMMA resist and fabricate Ga, Ag, and Au thin-film gratings.

After fabricating thin-film gratings, metal films were heated using a furnace under a nitrogen atmosphere to fabricate Ga, Ag, and Au MNHGs from the Ga, Ag, and Au thin-film gratings. The heating conditions were set at 300 °C for 10 min for Ga and Ag and 650 °C for 30 min for Au. These deposition and heating conditions were consistent with those used for the sample with metal nano-hemisphere structures across its entire surface, which was employed in the simulations. Additionally, to facilitate a comparison with Ag MNHG, Ag thin-film gratings with a thickness of 50 nm were also fabricated under the same conditions.

The MNHGs, fabricated as described, were subjected to white light from a LDLS light source (ENERGETIQ, EQ-99X-S-NA, Tokyo Instruments, Tokyo, Japan). Using a monochromator (M10-Monochromator, Bunkoukeiki Co., Ltd., Tokyo, Japan), light in 50 nm increments from 250 nm to 800 nm was directed onto the MNHG and the Ag thin-film gratings. The transmitted light intensity and the first-order diffracted light intensity were detected using a detector (Hamamatsu Photonics, C10027-01, Shizuoka, Japan). The diffraction efficiency *κ* was calculated from the ratio of these intensities.

## 3. Results and Discussion

**Figure 4:**
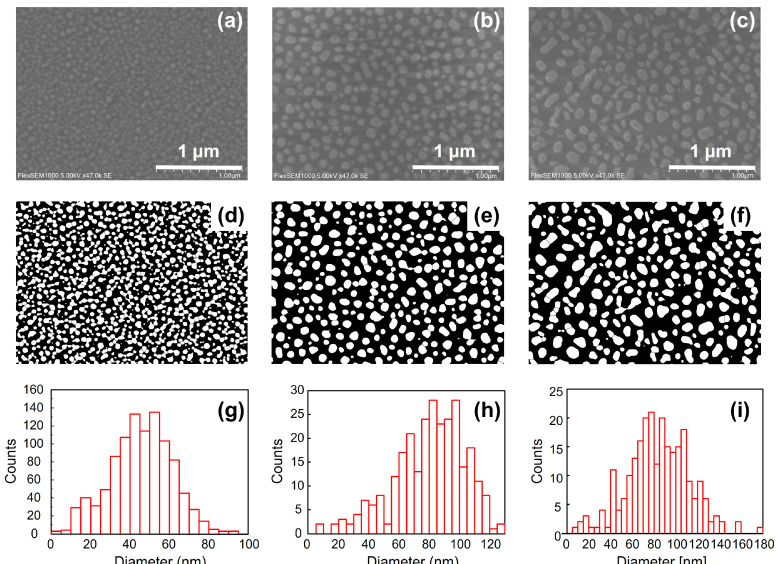
(**a**–**c**) present SEM images of Ga, Ag, and Au nano-hemisphere structures deposited on substrates without grating patterns, highlighting the nanoscale morphology of each metal. (**d**–**f**) show binarized versions of these images, facilitating the analysis of grain sizes. The histograms in (**g**–**i**) provide a statistical distribution of grain sizes for Ga, Ag, and Au nano-hemispheres, respectively. These results illustrate the variations in size and distribution of the nano-hemispheres, which are critical for understanding their localized surface plasmon resonance (LSPR) properties.

Figure 4a–c are scanning electron microscopy (SEM) images of Ga, Ag, and Au nano-hemisphere structures created on the front surface of the substrate without a grating pattern. In all cases, nano-sized grain structures are observed. Figure 4d–f are binarized images of the SEM images in Figure 4a–c obtained through image analysis. From these binarized images, the respective grain sizes were analyzed and displayed in the histograms in Figure 4g–i. The sizes of the formed nanoparticles varied irregularly, making them difficult to optimize using Gaussian or lognormal distribution functions. The average grain sizes of Ga, Ag, and Au nano-hemispheres are 46 nm, 81 nm, and 84 nm, respectively, with an area density of 39%, 33%, and 33%, respectively. Although the nano-hemispheres were fabricated under the same conditions, their sizes and densities vary depending on the type of metal. The size and density of the nano-hemispheres significantly influence their LSPR optical properties and need to be analyzed and controlled in detail in the future. 

As observed in these SEM images, the nanostructures fabricated in this study are arranged randomly. Unlike photonic crystals, where the periodic structure is crucial for forming photonic bands and controlling optical properties, the optical properties of metal nanostructures driven by LSPR are largely independent of the periodicity of the structure. Each metal nano-hemisphere interacts with light individually, and it is well understood that the overall optical properties are minimally influenced by the periodic arrangement of the nanostructures [22].

There have been many reports on such process-fabricated metal nanostructures for a variety of metals, and the three species used in this study have been well studied. Ag, Au, and Ga nanoparticles exhibit distinct shape and surface oxidation characteristics when formed through thin-film deposition followed by thermal treatment. When Ag thin films are subjected to thermal treatment, they tend to aggregate due to surface energy minimization, forming nanoparticles that are typically spherical in shape [1]. The morphology of these nanoparticles can vary depending on the specific thermal conditions, such as the temperature and duration of the heat treatment [2]. Surface oxidation is relatively common for silver nanoparticles, especially when treated in an oxygen-rich environment, leading to the formation of a thin layer of silver oxide (Ag_2_O) on the surface [1]. Au thin films, when thermally treated, also form spherical nanoparticles due to similar surface energy considerations [23]. However, gold is much more chemically stable than silver, and as a result, gold nanoparticles generally do not undergo significant surface oxidation under typical experimental conditions [24]. The stability of gold against oxidation is one of the reasons why it is widely used in applications requiring long-term chemical resistance. Ga, with its low melting point, behaves differently from silver and gold. When gallium thin films are thermally treated, the metal tends to form liquid-like droplets that solidify into nanoparticles upon cooling [25]. These nanoparticles are typically spherical or elliptical, depending on the cooling rate and ambient conditions [25]. However, unlike gold, gallium is highly prone to oxidation, and a thin layer of gallium oxide (Ga_2_O_3_) forms on the surface of the nanoparticles almost immediately upon exposure to air [26]. This oxide layer can significantly affect the optical and electronic properties of the nanoparticles. Therefore, it is highly likely that the metal nanoparticles fabricated in this study also have oxidized surfaces and may exhibit deformed shapes from a hemispherical structure. To thoroughly analyze these aspects, it would be necessary to observe the cross-sectional shapes using TEM or SEM and to perform elemental analysis with EDX or similar techniques. While these detailed analyses are essential for future work, the primary objective of this study was to fabricate diffraction gratings with LSPR characteristics. We have confirmed that all three metals exhibit favorable LSPR properties, and thus, a more detailed analysis involving shape and composition will be addressed in subsequent studies.

**Figure 5:**
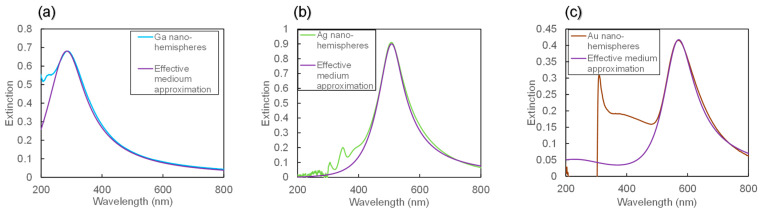
Extinction spectra for Ga (**a**), Ag (**b**), and Au (**c**) nano-hemispheres, as obtained from both experimental measurements and effective medium approximation (EMA) simulations. The plots compare the experimental extinction spectra with the spectra predicted by the EMA model, showing good agreement for each type of metal. The peaks observed in the extinction spectra correspond to the localized surface plasmon resonance (LSPR) wavelengths of each metal, with Ga peaking around 300 nm, Ag around 500 nm, and Au around 570 nm. These results confirm the occurrence of LSPR in the nano-hemisphere structures.

Figure 5a–c show the extinction spectra of Ga, Ag, and Au nano-hemisphere structures, along with the reproduced attenuation spectra using the EMA. The extinction spectra reproduced through the EMA closely match the peaks of the experimental attenuation spectra. Notably, the attenuation spectra for Ga, Ag, and Au nano-hemisphere structures exhibit peaks around wavelengths of 300 nm, 500 nm, and 570 nm, respectively. The extinction is suppressed at wavelengths other than these peaks, indicating the occurrence of LSPR for each metal nano-hemisphere. The extinction spectrum of Au nanoparticles consists of two primary components [1,2]. The first is the contribution from LSPR, which occurs in the red wavelength region, typically around 500–600 nm. The second component is related to the electronic transitions within the Au atoms themselves, particularly in the short-wavelength region below 500 nm. These transitions involve the excitation of electrons from the d-band to the sp-band of gold, which contributes to the absorption in this region of the spectrum. The EMA spectrum in Figure 5 deviates from the experimental spectrum at wavelengths shorter than 500 nm because it only accounts for the LSPR component.

The parameters (*ε*_∞_, *ε*_b_, *γ*, *ω*^2^, *ω*_p_^2^) used for the EMA were set as follows: for Ga (*ε*_∞_ = 1.5, *ε*_b_ = 1, *γ* = 2.70 × 10^14^ [rad/s], *ω*^2^ = 4.00 × 10^31^ [rad^2^/s^2^], *ω*_p_^2^ = 7.00 × 10^31^ [rad^2^/s^2^], for Ag (*ε*_∞_ = 2.5, *ε*_b_ = 1, *γ* = 5.35 × 10^14^ [rad/s], *ω*^2^ = 1.34 × 10^31^ [rad^2^/s^2^], *ω*_p_^2^ = 2.30 × 10^31^ [rad^2^/s^2^]), and for Au (*ε*_∞_ = 3.5, *ε*_b_ = 1, *γ* = 6.00 × 10^15^ [rad/s], *ω*^2^ = 1.09 × 10^31^ [rad^2^/s^2^], *ω*_p_^2^ = 9.40 × 10^30^ [rad^2^/s^2^]). 

**Figure 6:**
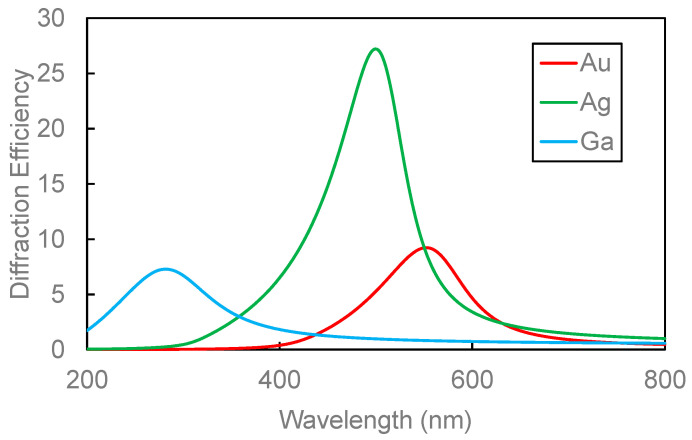
Simulated diffraction efficiencies of metal nano-hemisphere gratings (MNHGs) made from Ga, Ag, and Au. The graph shows how each material’s nano-hemisphere structure influences the diffraction efficiency across different wavelengths. The diffraction peaks correspond to the localized surface plasmon resonance (LSPR) of each metal, with Ga peaking around 300 nm, Ag near 500 nm, and Au at approximately 570 nm. These results highlight the wavelength-selective diffraction capabilities of the MNHG structures.

Figure 6 shows the simulation results of diffraction efficiency of the Ga, Ag and Au MNHGs calculated by these parameters. The diffraction efficiency of Ga, Ag, and Au MNHGs exhibits peaks around wavelengths of 300 nm, 500 nm, and 570 nm, respectively, with lower values at wavelengths other than the peaks. The positions of these peaks closely align with the peak positions of the extinction spectra due to LSPR in each metal nano-hemisphere, suggesting the emergence of peaks in the diffraction efficiency due to LSPR. From these results, it can be estimated from the simulation outcomes that MNHGs strongly diffract light near the resonance wavelength of the LSPR for each metal, while light at other wavelengths is not diffracted.

**Figure 7:**
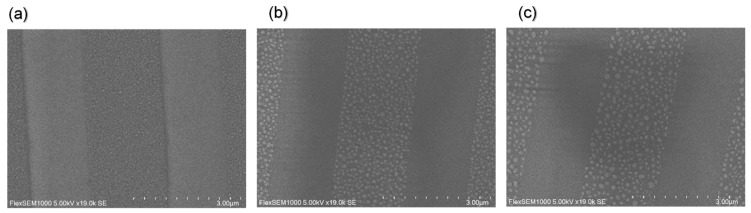
Scanning electron microscope (SEM) images of the fabricated metal nano-hemisphere grating (MNHG) structures with Ga (**a**), Ag (**b**), and Au (**c**). These images confirm the successful fabrication of MNHGs, showing metal nano-hemispheres arranged in a regular grating pattern. The images also highlight the distinct sizes and morphologies of the nano-hemispheres for each metal, which contribute to their unique optical properties.

Figure 7a–c show the scanning electron microscope (SEM) images of Ga, Ag, and Au MNHGs, respectively. The successful fabrication of gratings with metal nano-hemisphere structures arranged in a pattern is confirmed. These images confirm the successful fabrication of the MNHGs. From Figure 7, it can be observed that the sizes of Ga, Ag, and Au nano-hemisphere structures are approximately 60–80 nm, 120–160 nm, and 60–100 nm, respectively.

**Figure 8:**
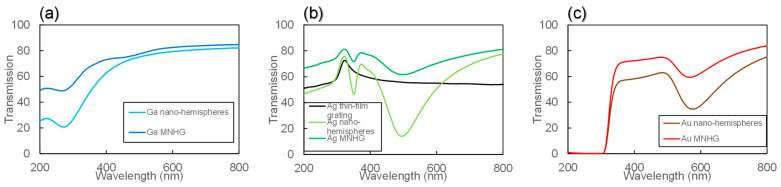
Transmission spectra of metal nano-hemisphere grating (MNHG) structures fabricated with Ga (**a**), Ag (**b**), and Au (**c**). The graphs show the transmission behavior of each MNHG across the visible spectrum, highlighting the characteristic decrease in transmission near the wavelength corresponding to the extinction peaks seen in Figure 5. Specifically, the Ag MNHG shows a marked reduction in transmission near 500 nm, which corresponds to the localized surface plasmon resonance (LSPR) of the silver nano-hemisphere structure. Similarly, the Ga and Au MNHGs exhibit transmission dips near their respective LSPR wavelengths. These results confirm the strong wavelength-selective behavior of the MNHG structures.

Figure 8a–c show graphs of the transmission for Ga, Ag, and Au MNHGs, along with a sample featuring nano-hemisphere structures across its entire surface. Figure 8b also includes the transmission of the Ag thin-film grating. The Ag thin-film grating exhibits a nearly constant transmission in the visible light range (450 nm to 800 nm). On the other hand, the Ag MNHG shows a decrease in transmission near 500 nm, corresponding to the wavelength where the extinction peak was observed in the extinction spectrum in Figure 5b. This decrease in transmission is believed to be caused by the localized surface plasmon resonance of the silver nano-hemisphere structure. Similarly, in Figure 8a,c, a decrease in transmission is observed near the wavelength corresponding to the extinction peak in the extinction spectrum. These decreased peaks are considered to be induced by the LSPR of Ga or Au nano-hemisphere structures.

**Figure 9:**
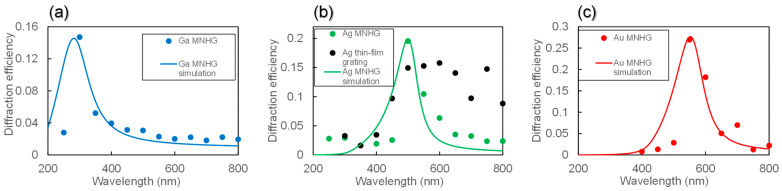
Measured diffraction spectra of metal nano-hemisphere grating (MNHG) structures made from Ga (**a**), Ag (**b**), and Au (**c**). The solid lines represent the simulation results of the diffraction efficiency for each MNHG. The comparison between the experimental and simulated results shows good agreement, indicating the wavelength-selective diffraction characteristics of the MNHGs. The diffraction efficiency for the Ag MNHG shows a peak around 500 nm, which is consistent with the resonance wavelength, while the diffraction efficiency for the Ag thin-film grating remains nearly constant across the visible range. These results confirm that the MNHG structures selectively diffract light near the resonance wavelength influenced by the localized surface plasmon resonance (LSPR) of the nano-hemisphere structure.

Figure 9a–c show the measured diffraction efficiency of Ga, Ag, and Au MNHGs, with solid lines representing the simulation results of the diffraction efficiency for each MNHG, as shown in Figure 6. Additionally, Figure 9b includes the diffraction efficiency of the Ag thin-film grating. The experimental results were consistent with the calculated trends and peak positions. From Figure 9b, it can be observed that the diffraction efficiency of the Ag thin-film grating remains nearly constant in the visible light range (500 nm to 800 nm). Comparing this with the transmission spectra in Figure 8b, the diffraction efficiency remains constant in the wavelength range where the transmission is constant (450 nm to 800 nm), and decrease in the wavelength range where the transmission is high (300 nm to 400 nm). This suggests that the diffraction efficiency of the Ag thin-film grating is consistent with the transmission data, indicating its action as a diffraction grating in the entire visible light range. On the other hand, the diffraction efficiency of the Ag MNHG reaches its maximum around 500 nm, and at other wavelengths, the diffraction efficiency is low. This result is consistent with the simulation results and transmission data. 

**Figure 10:**
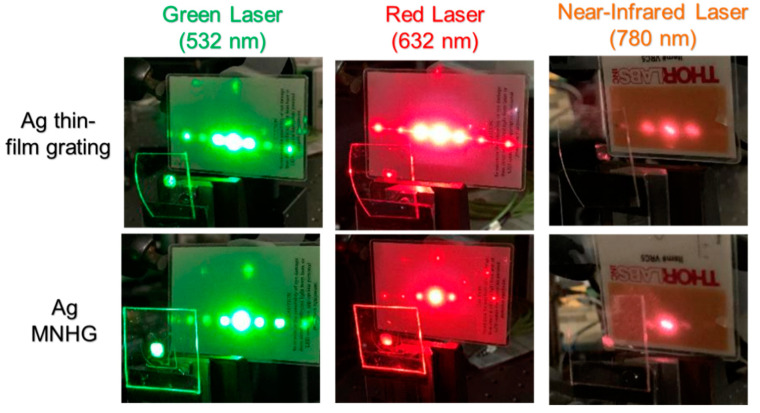
Diffraction spectra of thin-film grating and metal nano-hemisphere grating (MNHG) structures fabricated with he results demonstrate that while the Ag MNHG strongly diffracts green laser light, it exhibits minimal diffraction with near-infrared light, consistent with its wavelength-selective properties due to localized surface plasmon resonance (LSPR). These findings confirm that each MNHG selectively diffracts light near its resonance wavelength.

Figure 10 shows photographs of the diffraction patterns when laser light is irradiated onto the Ag MNHG and the Ag thin-film grating. From these results, it is confirmed that the Ag thin-film grating diffracts laser light at any wavelength, while the Ag MNHG strongly diffracts green laser light and almost does not diffract near-infrared laser light. This result is consistent with the findings in Figure 9, confirming that the Ag MNHG exhibits wavelength selectivity.

Based on these results, it has been confirmed that MNHG selectively diffracts light near the resonance wavelength influenced by LSPR of the nano-hemisphere structure. Utilizing these results, it will be possible to fabricate a diffraction grating that diffracts pump light while transmitting probe light, enabling the construction of a novel third-order nonlinear laser spectroscopy method, as described in the Introduction. This approach allows for high-sensitivity, low-noise, and stable measurements with a simple and straightforward setup, making it possible to selectively observe a wide range of phenomena, including electron dynamics [27], molecular dynamics [28], thermal dynamics [29], and chemical reactions [30]. We anticipate that this will expand the applications of third-order nonlinear spectroscopy across various fields. Moreover, beyond its use in spectroscopy, fabricating MNHGs on the surfaces of different materials could significantly enhance their nonlinear optical effects, opening new avenues for material design and functionalization. This ability would lead to stronger nonlinear optical signals, facilitating the detection and study of effects such as multi-photon absorption [31], harmonic generation [32], and nonlinear refractive index changes [33]. This enhancement allows for achieving the same nonlinear effects with lower input light power, which is especially beneficial in sensitive experimental setups. Furthermore, by adjusting the metal type, size, and shape of the nano-hemispheres, the LSPR wavelength and field enhancement can be tailored, enabling the design of materials with specific nonlinear optical properties at desired wavelengths. Overall, incorporating MNHGs on material surfaces is a promising approach to significantly boosting nonlinear optical effects, paving the way for new applications in nonlinear optics, photonics, and optical sensing.

## 4. Conclusions

In this paper, we investigated the optical properties of the MNHG, where Ga, Ag, and Au nano-hemispheres were arranged in a grating pattern. The results demonstrated that Ga, Ag, and Au MNHGs selectively diffracted light near wavelengths of 300 nm, 500 nm, and 570 nm, respectively, while transmitting light at other wavelengths, showcasing wavelength selectivity. Furthermore, it was confirmed that the wavelength selectivity was attributed to the LSPR induced by each metal nano-hemisphere. When applying these results to the MPT-TG method, it would become possible to diffract only the pump light using the MNHG, while the probe light will be diffracted solely through the third-order nonlinear polarization. This suggests the potential to construct a highly sensitive third-order nonlinear laser spectroscopy method by reducing background signals and thereby enhancing detection sensitivity. Consequently, this advancement could enable the detection of subtle changes that were previously challenging to observe, expanding the capability to investigate the characteristics of a wide range of substances.

## Data Availability

The data presented in this study are available on request from the corresponding author.
